# CA19-9 as a prognostic factor in inoperable pancreatic cancer: the implication for clinical trials

**DOI:** 10.1038/sj.bjc.6602760

**Published:** 2005-09-20

**Authors:** N R Maisey, A R Norman, A Hill, A Massey, J Oates, D Cunningham

**Affiliations:** 1 Department of Medicine, Royal Marsden Hospital, Downs Road, Sutton, Surrey SM2 5PT, UK

**Keywords:** pancreatic cancer, CA19-9, prognosis, chemotherapy

## Abstract

In a multivariate analysis of 154 patients receiving chemotherapy, baseline CA19-9 was an independent prognostic factor for overall survival (OS) (HR 1.8; 95% CI: 1.3–2.5, *P*=0.0004). The 1-year OS was 19 and 46%, respectively, for patients with a baseline CA19-9 above or below the median value. A fall of 20% in CA19-9 level from baseline was an independent prognostic factor for OS (HR 1.9; 95% CI: 1.1–3.4, *P*=0.019).

Over the last decade, systemic chemotherapy has become a standard therapy for patients with inoperable pancreatic cancer. Prior to this, the lack of randomised data and the perceived toxicity of treatment led most clinicians to believe that cytotoxic therapy would not be of palliative benefit or could significantly prolong their patients' lives. Following a number of randomised clinical trials, it became clear that not only did chemotherapy offer a small but significant survival benefit, but there was also evidence supporting an improvement in quality of life. Over the last decade, a number of relatively large and well-conducted phase III trials have attempted to define the optimal chemotherapy schedule for this cohort of patients. Currently, the standard of care is gemcitabine, following the seminal paper by Burris *et al* (1997). A number of ongoing trials are exploring the potential benefit of gemcitabine scheduling, combination with other chemotherapeutic agents and the addition of novel agents such as antibodies to the epidermal growth factor receptor and vascular endothelial growth factor.

In the mid 1990s, the CONSORT (Consolidated Standards of Reporting Trials) statement was published, which was intended to improve the quality of reporting of phase III trials (Moher *et al*, 2001). The CONSORT statement consists of a checklist and a diagram intended for use in writing, reviewing or assessing reports of randomised controlled trials. One of the items in the checklist is the reporting of baseline demographic and clinical characteristics of each group.

When assessing the results of a phase III trial, it is clearly vital that the known important prognostic factors are balanced between treatment arms to allow a fair comparison of treatment effect. Despite a small number of publications suggesting that the tumour marker CA19-9 is an important prognostic variable (Gogas *et al*, 1998; Halm *et al*, 2000; Saad *et al*, 2002; Stemmler *et al*, 2003; Ziske *et al*, 2003), none of the large published randomised studies of chemotherapy to date have reported baseline CA19-9 levels.

The aim of this retrospective analysis was to investigate whether CA19-9 provided prognostic information in patients with inoperable pancreatic cancer treated with systemic chemotherapy, and should therefore be reported in future randomised controlled trials.

## MATERIALS AND METHODS

All patients treated with gemcitabine- and/or 5-fluorouracil-based chemotherapy in three consecutive phase III randomised trials at this centre were eligible for analysis. Patients had inoperable or metastatic histologically proven pancreatic cancer, were of WHO performance status (PS) 0–2 and had radiologically measurable disease. All patients were previously untreated. Entry criteria included a total serum bilirubin level of 30 *μ*mol l^−1^ or less. Informed written consent was obtained from all patients.

### Treatment

All eligible patients received one of four chemotherapy regimens. Protracted venous infused 5-fluorouracil (PVI 5FU) was either given alone (at a dose of 300 mg m^−2^ day^−1^ for a maximum of 24 weeks) or in combination with mitomycin C (MMC) administered at a dose of 7 mg m^−2^ 6 weekly for four courses. The remainder received a combination of gemcitabine (1000 mg m^−2^ on days 1, 8 and 15 of a 28-day cycle) and capecitabine (830 mg m^−2^ twice daily for 21 days followed by a week's break) or gemcitabine alone, at a dose of 1000 mg m^−2^ weekly for 7 weeks, followed by a week's break and subsequently for 3 weeks out of every 4.

### Measurements

Serum CA19-9 was measured at baseline (defined as being within 30 days before the start of chemotherapy) and subsequently every 6 weeks while the patients remained on treatment. The CA19-9 concentration was measured by an automated, commercially available enzyme immunoassay on an axsym analyser (Abbott Diagnostics Laboratory). A value of 37 U ml^−1^ was used as the upper limit of normal.

### Statistics

All data were recorded prospectively on the Gl unit database. Overall survival (OS) (defined as time from randomisation to death from any cause) was examined with the Kaplan–Meier product limit method (Kaplan and Meier, 1958), and survival in patients with baseline CA19-9 values that fell above or below the median level (median dichotomised) was compared with the log-rank test (Peto and Peto, 1972). Statistical analysis was performed using the statistical package SPSS for Windows version 12.01. Multivariate logistic regression was used to determine factors predictive of survival. Factors included in these analyses were baseline CA19-9, a 20% drop in CA19-9 level following the start of chemotherapy, age, sex, the presence of locally advanced or metastatic disease and performance status. A fall of 20% in the CA19-9 level was chosen after review of similar analyses in the literature. *P*-values of less than 0.05 were considered statistically significant.

## RESULTS

### Patients

Between July 1994 and July 2004, 218 patients received chemotherapy in the context of a clinical trial. Of these patients, 154 had a CA19-9 level recorded within 30 days before the start of chemotherapy at this centre. Baseline demographics are given in Table 1.

### CA19-9 levels

Baseline CA19-9 levels were recorded in 154 patients. The median time of performing the test was 1 day prior to the start of chemotherapy (range 0–30). The median baseline CA19-9 level was 958 U ml^−1^.

Post-treatment CA19-9 was recorded in 88 patients. The median time of performing the test from the start of chemotherapy was 42 days, ranging from 28 to 55 days. The median post-treatment CA19-9 level was 998 U ml^−1^. There was no significant difference in the time to progression (TTP) between the cohort that had CA19-9 levels performed at baseline and following chemotherapy, compared with the cohort that had baseline levels only (*P*=0.46).

### Survival

**Figure 1:**
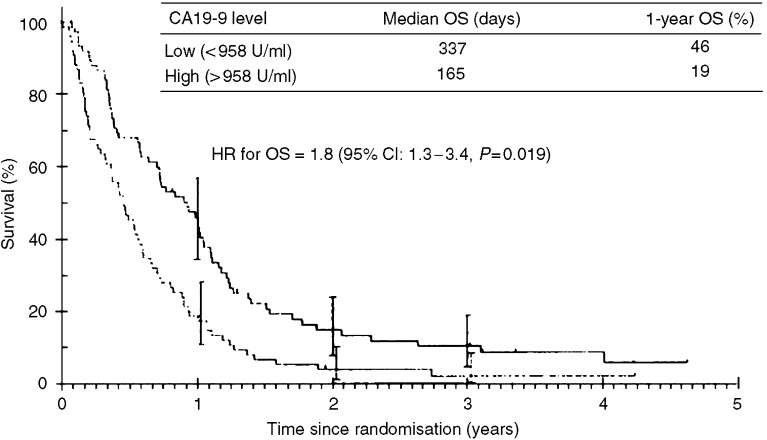
Overall survival and pre-treatment CA19-9 level.

The median follow-up in those patients with a recorded baseline CA19-9 level was 712 days and 92% had died at the time of analysis. The median OS in patients with a baseline CA19-9 below the median value was significantly superior to those with a value above (337 *vs* 165 days, *P*=0.0004). The 1-year OS was 19 and 46%, respectively, for patients with a baseline CA19-9 above or below the median value (see Figure 1).

In a multivariate analysis, baseline CA19-9 above or below the median value (958 U ml^−1^) was found to be an independent prognostic factor for OS (HR 1.8; 95% CI: 1.3–2.5, *P*=0.0004).

A fall of 20% in CA19-9 level at first measurement following the start of treatment was an independent prognostic factor for OS (HR 1.9; 95% CI: 1.1–3.4, *P*=0.019). Baseline characteristics in this group were not significantly different from the original cohort.

Other factors that were found to be independent prognostic factors for OS were sex and performance status (PS) (see Table 2).

## DISCUSSION

Ensuring an equal balance of prognostic factors between treatment arms in randomised controlled trials is vital. This may be particularly relevant when the clinical benefit of an intervention is likely to be small, as is the case for patients with advanced pancreatic cancer. A number of both patient- and disease-related factors have been identified as independent prognostic markers, including PS, sex and the presence of metastases, and are routinely quoted in clinical studies as baseline characteristics. There have also been several reports of various biological markers that may carry some prognostic significance. These include growth factors such as vascular endothelial growth factor and platelet-derived endothelial cell growth factor (Fujimoto *et al*, 1998), microsatellite instability (Nakata *et al*, 2002), tumour suppression gene expression such as SMAD4 (Biankin *et al*, 2002) and the expression of mutated genes controlling response to DNA damage, such as GADD45a and p53 (Yamasawa *et al*, 2002).

The serum carbohydrate antigen CA19-9 is a tumour-associated antigen that has been shown to be a highly specific and sensitive serum marker for pancreatic cancer (Pleskow *et al*, 1989; Rollhauser and Steinberg, 1998). CA19-9 is a sialylated Lewis blood group antigen targeted by the monoclonal antibody 1116 NS 19-9 (Koprowski *et al*, 1979). Previous studies have demonstrated a prognostic role for pretreatment CA19-9 in patients receiving radiotherapy (Katz *et al*, 1998) or undergoing pancreatic resection (Glenn *et al*, 1988). Although a small number of papers have described a fall in CA19-9 to be an independent prognostic variable for survival (Gogas *et al*, 1998; Saad *et al*, 2002; Stemmler *et al*, 2003; Ziske *et al*, 2003), there have been surprisingly few studies investigating the use of baseline CA19-9 in predicting survival for patients with inoperable pancreatic cancer who undergo systemic chemotherapy. Halm *et al* (2000) reported the survival of 43 consecutive patients with advanced pancreatic cancer treated with single-agent gemcitabine. In a multivariate analysis, baseline CA19-9 was reported to be an independent prognostic predictor of survival with a relative risk of death 1.4 (95% CI: 1.0–2.0, *P*=0.04). Ziske *et al* (2003) published their results in a similar cohort of patients (*n*=46). Although they did not report baseline CA19-9 level as a prognostic variable, they did find that a fall of at least 20% in CA19-9 level following the start of chemotherapy was the only independent prognostic marker for OS in their Cox multivariate analysis. An important caveat in the interpretation of CA19-9 levels in pancreatic cancer is the presence of biliary obstruction, which results in the elevation of the marker level. In the current series, however, entry criteria mandated that serum bilirubin should be 30 *μ*mol l^−1^ or less, and therefore this potential pitfall was avoided in the interpretation of baseline CA19-9 levels.

The results of this study suggest that baseline tumour marker CA19-9 is an important independent prognostic variable in patients with inoperable pancreatic cancer. We found that baseline CA19-9 above or below the median value (958 U ml^−1^) was an independent prognostic factor for OS (HR 1.8; 95% CI: 1.3–2.5, *P*=0.0004), with a 1-year OS of 19 and 46%, respectively. Although this is the largest reported series examining the predictive value of baseline CA19-9 in patients treated with systemic chemotherapy, it is still a relatively small cohort of 154 patients, and further studies would be welcome to consolidate these results. However, considering its apparent prognostic value, future randomised controlled trials should consider reporting median CA19-9 levels in treatment groups as part of routine demographic data.

## Figures and Tables

**Table 1:** Baseline patient characteristics

**Characteristic**	**Total patient cohort**	**Patients with baseline CA19-9**	**Patients with pre- and post-treatment CA19-9**
*n*	218	154	88
Median age (range) (years)	63 (28–86)	63 (28–86)	63 (42–86)
Male (%)	62.8	63.6	62.5
			
*Disease extent* (%)			
Metastatic	62.8	66.2	64.7
Locally advanced	37.2	33.8	35.3
			
*Performance status* (%)			
0	12.8	12.9	17.0
1	56.9	62.3	62.5
2	25.7	24.7	20.5
3	0.9	—	—
Not documented	3.7	—	—
			
*Chemotherapy received* (%)			
None	4	0	0
PVI 5FU	48	51	45.5
PVI 5FU+MMC	33	28	21.6
GEM	6	8.5	19.4
GEM+CAP	9	12.5	12.5
Other	0	0	1

PVI 5FU=protracted venous infused 5-fluorouracil, MMC=mitomycin C, GEM=gemcitabine, CAP=capecitabine.

**Table 2:** Results of the multivariate analysis

**Variable**	**Hazard ratio**	**95% confidence interval**	***P*-value**
*Baseline CA19-9*			
Above *vs* below median	1.84	1.31–2.57	0.0004
			
*CA19-9 Response*			
<20% drop *vs* ⩾20% drop	1.95	1.11–3.42	0.019
			
*PS*			
2–4 *vs* 0–1	1.75	1.19–2.57	0.004
			
*Sex*			
Male *vs* female	1.52	1.06–2.16	0.02
